# Metastatic Hepatocellular Carcinoma Masquerading as Acute Diverticulitis

**DOI:** 10.14309/crj.0000000000000913

**Published:** 2022-11-24

**Authors:** Tim Brotherton, Ahmad M. Al-Taee, Danielle Carpenter, Antonio R. Cheesman

**Affiliations:** 1 Department of Internal Medicine, Saint Louis University Hospital, St. Louis, MO; 2 Department of Gastroenterology and Hepatology, NYU Langone Health, St. Louis, MO; 3 Department of Pathology, Saint Louis University Hospital, St. Louis, MO; 4 Department of Gastroenterology and Hepatology, Saint Louis University Hospital, St. Louis, MO

## Abstract

Colorectal cancer may masquerade as acute diverticulitis. Our case is a 71-year-old man who presented to the emergency department with abdominal pain and was diagnosed with acute diverticulitis. He was ultimately found to have metastatic hepatocellular carcinoma to the colon without any evidence of diverticular disease on colonoscopy. Although the most common malignancy to masquerade as diverticulitis is colorectal cancer, metastatic deposits should also be considered, especially in patients with a history of extracolonic malignancy.

## INTRODUCTION

Diverticulosis is a common condition, present in over 50% of Americans aged 60 years and older.^1^ Acute diverticulitis occurs in up to 5% of patients with diverticulosis, and of those who experience acute diverticulitis, approximately 20% will have a repeat episode.^2,3^ Cases are considered complicated when acute diverticulitis is accompanied by abscess, perforation, stricture, or fistula. A review in 2013 found underlying colorectal cancer in 1.6% of patients with acute diverticulitis.^4^ When compared with an age and sex-adjusted reference population, the risk of colorectal cancer after acute diverticulitis was found to be 44-fold higher.^5^

Guidelines recommend performing a colonoscopy 6–8 weeks after resolution of symptoms to rule out an underlying colonic malignancy. In this case report, we present a unique case of metastatic hepatocellular carcinoma (HCC) mimicking acute diverticulitis in a patient with a history of HCC that had been in remission.

## CASE REPORT

A 71-year-old man presented to the emergency department with a 1-week history of generalized abdominal pain and fatigue. Medical history included orthotopic liver transplantation performed 7 years earlier, for hepatitis C-related cirrhosis complicated by HCC. He was on tacrolimus for immunosuppression. Two years before, he had recurrence with metastasis to the left adrenal gland, which was resected. Notably, his HCC had been in remission on surveillance computed tomography and magnetic resonance imaging of his abdomen and pelvis, with his most recent examinations performed just 10 days before presentation.

**Figure 1.**
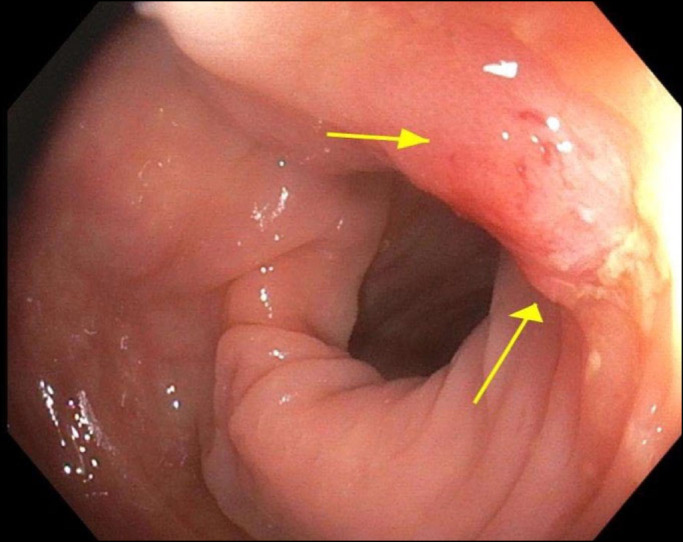
Descending colon with are of irregularity concerning for infiltrative lesion (yellow arrows).

**Figure 2.**
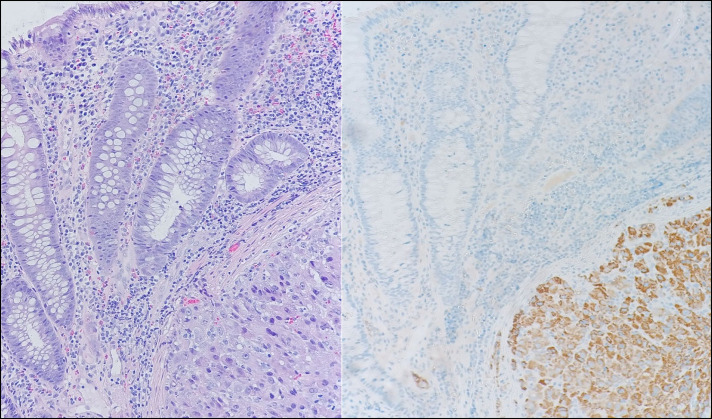
Descending colon at 40 cm. (A) A nest of polygonal cells with ample eosinophilic cytoplasm in the submucosa (hematoxylin and eosin, 400×). (B) Positive hepatocyte-specific antigen (400×).

In the emergency department, he was afebrile with normal vital signs. A complete blood count and comprehensive metabolic panel were within normal limits. Abdominal computed tomography with intravenous contrast revealed acute complicated diverticulitis of the descending colon with a 1.4 cm intramural abscess and 1.1 cm abscess adjacent to the colon. These findings were new compared with abdominal cross-sectional imaging obtained 10 days before. There were no liver lesions. He was hospitalized and managed conservatively with bowel rest, intravenous fluids, and antibiotics. After a few days, his symptoms improved and he was discharged home with oral antibiotics. Two months later, he underwent a colonoscopy showing a small area of subtle irregularity in the descending colon that was concerning for a possible infiltrative lesion (Figure 1). Notably, there was no evidence of diverticulosis on the examination. Biopsies of the area of concern revealed metastatic HCC (Figure 2). The patient was referred to medical oncology for consideration of systemic therapies.

## DISCUSSION

Extrahepatic metastases in patients with HCC are not rare and the most common locations include the lungs, lymph nodes, bones, and adrenal glands.^6^ Metastasis to other parts of the gastrointestinal (GI) tract is less common and previously estimated to affect ∼2% of patients with HCC.^7^ A recent review by Yu et al regarding metastasis in patients with HCC surmised that upper GI sites (eg, stomach, duodenum) are more likely locations of metastasis because these are subject to direct invasion by the primary tumor.^8^ Lower GI tract sites (eg, distal colon, rectum) otherwise are unlikely targets for metastasis because of their location far from direct invasion and downstream from portal venous flow.^8^ The most frequent sites of colonic involvement include the ascending and transverse colon, with only one case reported of distal colonic involvement. A case series of HCC with colonic metastasis by Yu et al found that the most common presenting finding was GI bleeding.^9^ None of the patients in this review manifested with findings of acute diverticulitis.

Most cases of acute diverticulitis are uncomplicated, with only an estimated 10% of patients presenting with complicated disease.^10^ Multiple reviews have found the risk of occult malignancy to be significantly higher in patients with complicated as opposed to uncomplicated diverticulitis. The American Gastroenterological Association recommends all patients with an episode of complicated diverticulitis (and those after a first episode of acute uncomplicated diverticulitis) to undergo colonoscopy 6–8 weeks after the resolution of symptoms.^11^ This recommendation is also supported by guidelines from the American Society of Colon and Rectal Surgeons.^12^

These guidelines exist primarily to exclude colorectal cancer. However, it is important to remember that other malignancies may also metastasize to the colon and masquerade as acute diverticulitis. Such lesions may appear as ill-defined and possibly subepithelial in origin, as in the case of our patient, contrary to the classical findings of primary colorectal cancer which the endoscopist may be looking for. Moreover, patients with a history of liver transplant are at increased risk of solid organ cancers, a component of which may be due to adverse effects of immunosuppressive drugs (eg, tacrolimus).^13^

In summary, we present a case of recurrent HCC with metastasis to the descending colon masquerading as a case of acute complicated diverticulitis. In patients with a history of extracolonic malignancies, metastatic deposits should be considered in the differential diagnosis of acute diverticulitis and colonoscopy should be performed after symptom resolution. This case also emphasizes the importance of adherence to established guidelines regarding follow-up colonoscopy for patients with acute diverticulitis.

## DISCLOSURES

Author contributions: T. Brotherton and AM Al-Taee wrote the initial draft of the report. D. Carpenter provided images and descriptions of pathology slides. AR Cheesman served as supervising physician on the case who reviewed and edited the manuscript before submission. T. Brotherton is the article guarantor.

Financial disclosure: None to report.

Informed consent was obtained for this case report.
